# Supplementary material for: Cdc42 couples septin recruitment to the axial landmark assembly via Axl2 in budding yeast
Source: J Cell Sci. 2023 Oct 9;137(5):jcs261080. doi: 10.1242/jcs.261080 (PMC10617600; doi:10.1242/jcs.261080)
Supplement: Supplementary information [file joces-137-261080-s1.pdf]

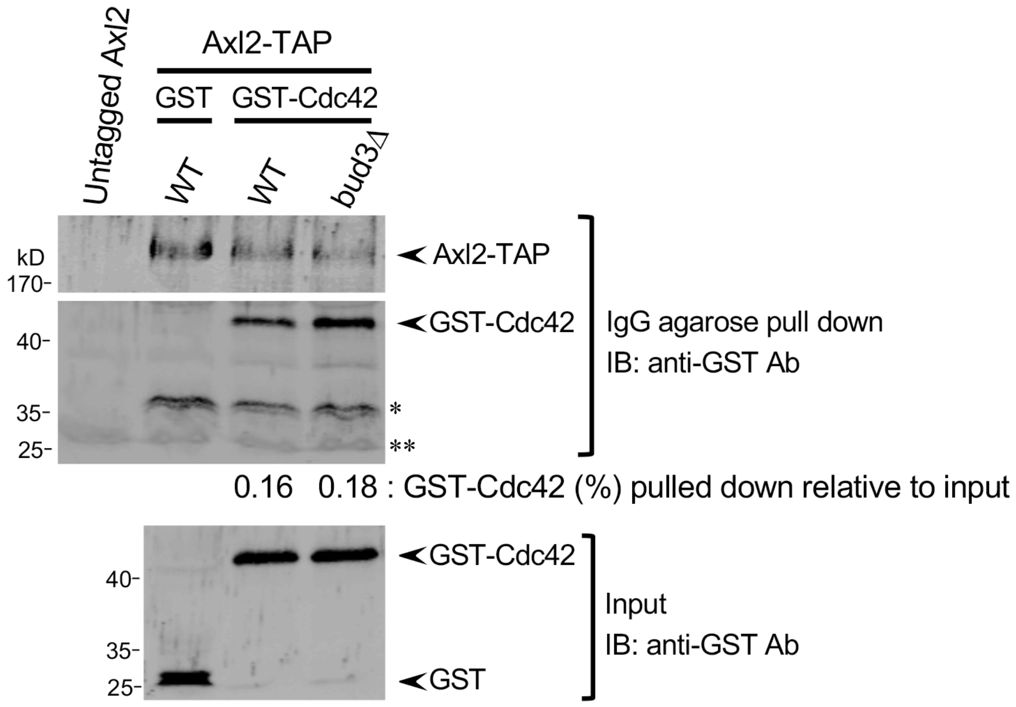

Fig. S1. Interaction between Cdc42 and Axl2 by TAP-pull-down assays.
Axl2-TAP was pulled down after incubating with GST-Cdc42 (or GST control) prepared from the *WT* and *bud3*Δ strains expressing Axl2-TAP. Lysates (input) and proteins pulled with IgG agarose were analyzed by immunoblotting with anti-GST antibodies, which also recognize the protein A tag in Axl2-TAP. GST-Cdc42 pull-down (%) shows the average amount recovered relative to input from two-independent protein preparations. The bands marked with * and ** are likely Axl2-TAP cleavage products and IgG light chain, respectively.

Journal of Cell Science • Supplementary information

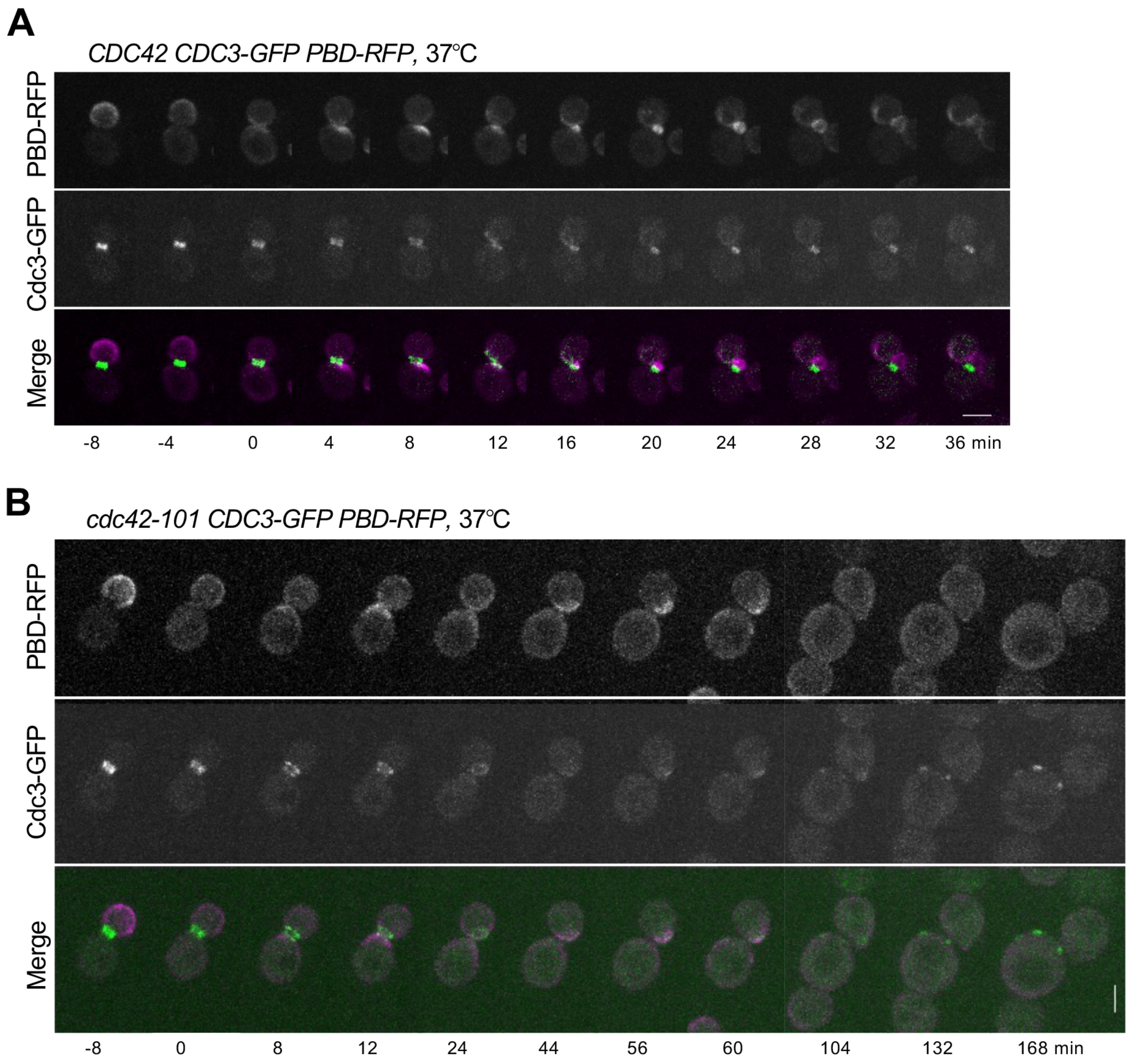

Fig. S2. Time-lapse images of WT and *cdc42-101* cells expressing Cdc3-GFP and PBD-tdTomato at 37°C

Images were captured every 4 min, starting at 1 h after shifting to 37°C from ~ 24°C. Images of WT (**A**) and *cdc42-101* (**B**) cells are shown at the selected time points. Time (min) is marked relative to the onset of cytokinesis (t = 0). Size bars, 3 µm.

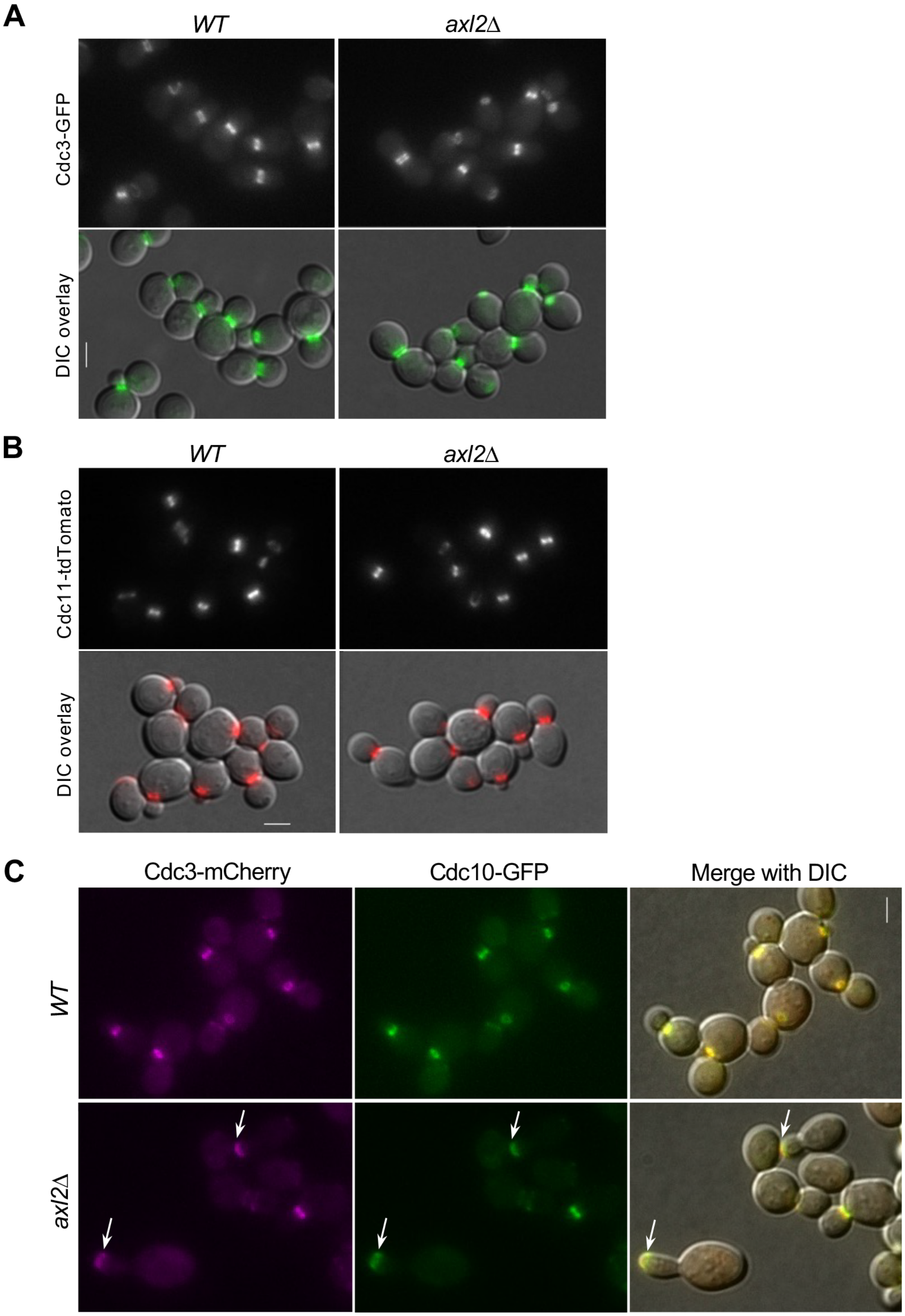

Fig. S3. Localization of septins in WT and mutant cells at 24°C.
A. Localization of Cdc3-GFP in WT and *axl2*Δ cells. Size bar, 3 µm.
B. Localization of Cdc11-tdTomato in WT and *axl2*Δ cells. Size bar, 3 µm.
C. Co-localization of Cdc3-mCherry and Cdc10-GFP in WT and *axl2*Δ cells. A small number of *axl2*Δ cells showed abnormal localization of both proteins to the bud tip (marked with arrows).

Journal of Cell Science • Supplementary information

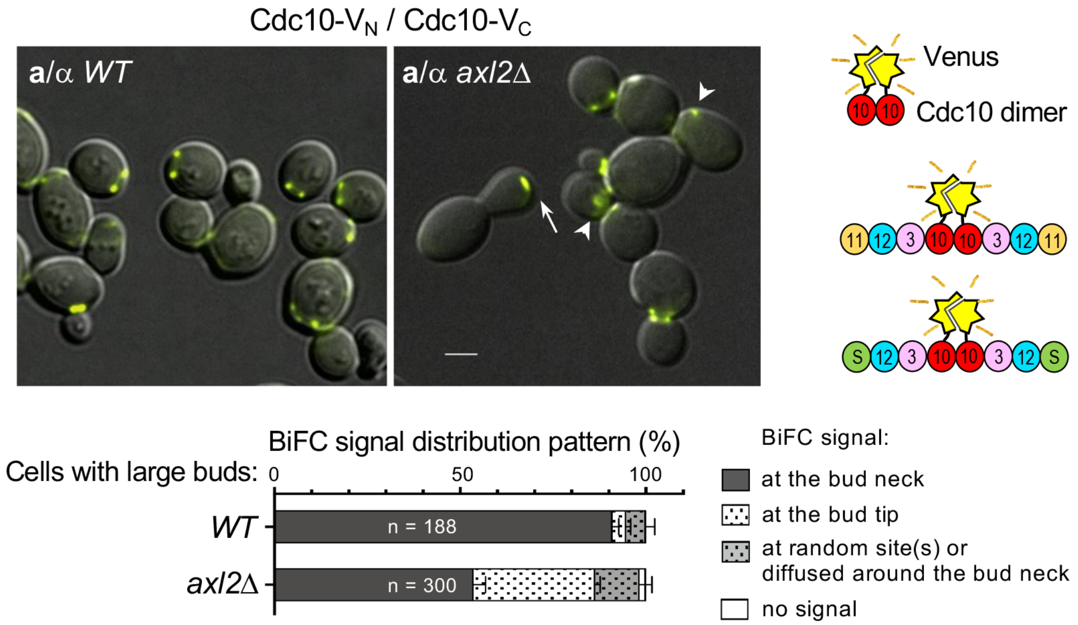

Fig. S4. BiFC assays with Cdc10-V_N and Cdc10-V_C in diploid WT and *axl2*Δ cells

The abnormal appearance of YFP signals in *axl2*Δ cells is marked with an arrow (at the bud tip) or arrowheads (diffused ring at the cell division site). Size bar, 3µm. The BiFC signal distribution patterns are analyzed from the total number (n) of cells (pulled from three imaging sets). Schemes (right) depict the possible reconstitution of Venus from the Cdc10 homodimer or Cdc10 dimers within the septin protofilaments.

Journal of Cell Science • Supplementary information

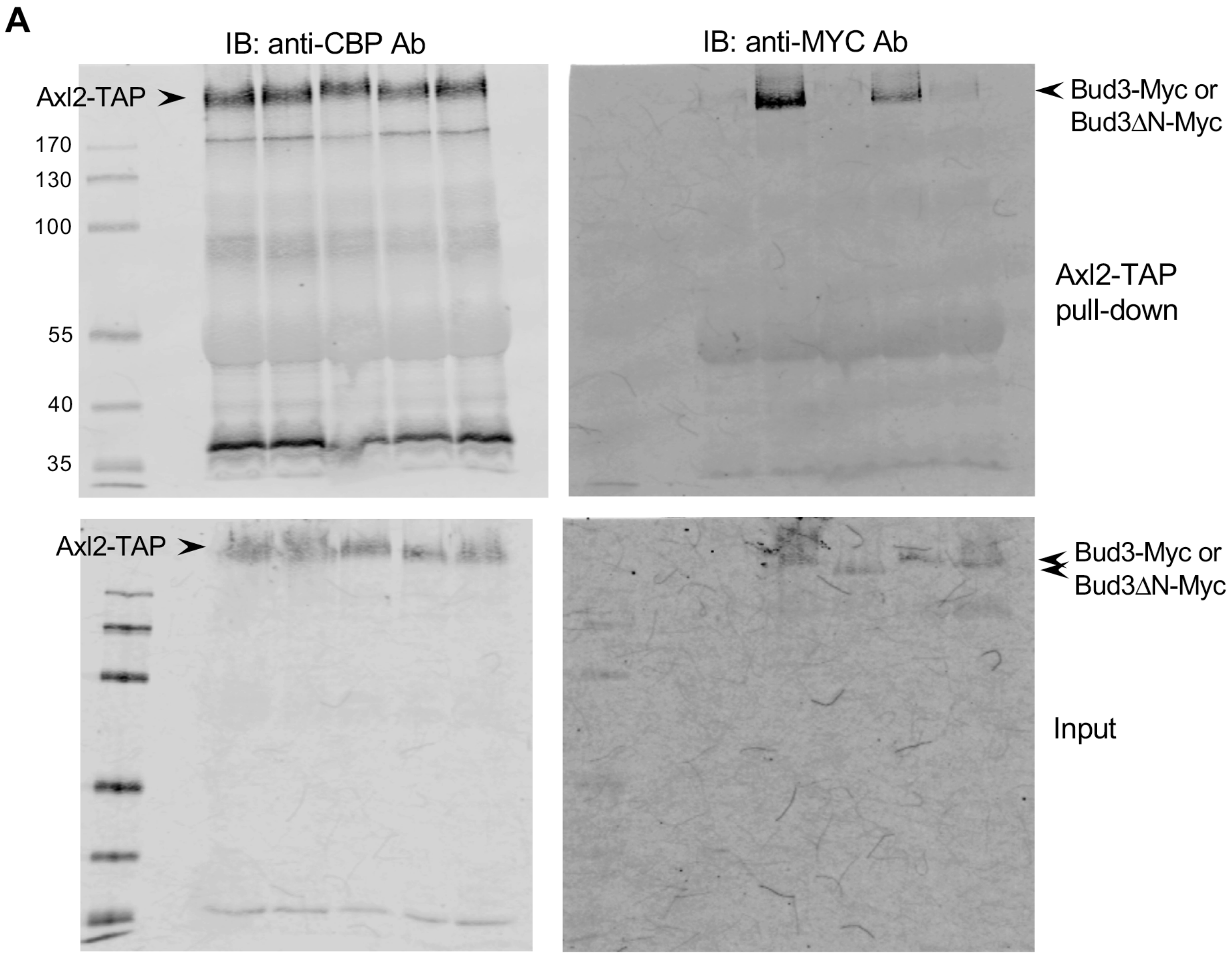

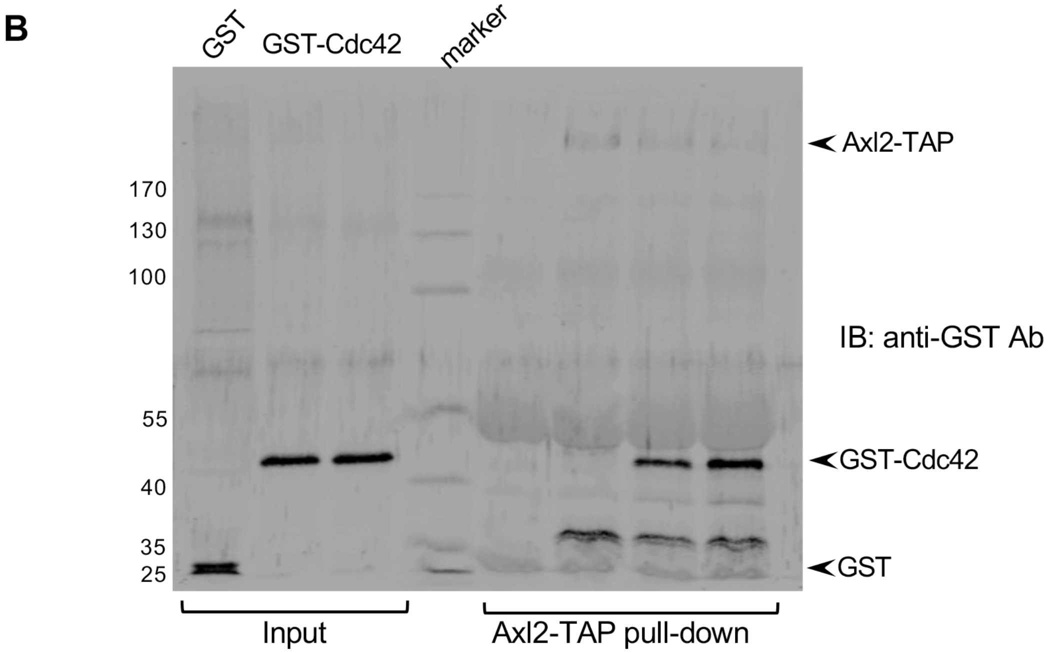

Fig. S5. Blot transparency

A. Axl2-TAP was detected using anti-CBP antibodies (two blots on the left), and Bud3-Myc was detected with anti-Myc antibodies (two blots on the right). See Fig. 1A.

B. GST-Cdc42 or GST was detected using anti-GST antibodies, which also recognize the protein A tag in Axl2-TAP. See Fig. S1.

Journal of Cell Science • Supplementary information

Table S1. Yeast strains used in this study

Strain [a]	Relevant Genotype	Source
DDY1301[@]	*MAT***a** *CDC42-LEU2 his3-Δ200 leu2-3, 112 lys2-801 ura3-52*	(Kozminski et al., 2000)
DDY1304[@]	*MAT***a** *cdc42-101(K5A)-LEU2*	(Kozminski et al., 2000)
EGY48	*MATα his3 trp1 ura3 LexA*$_{op(x6)}$*-LEU2*	(Gyuris et al., 1993)
HPY3657	*MATα his3 trp1 ura3 LexA*$_{op(x6)}$*-LEU2 bud3Δ::URA3*	This study
YEF473A[#]	*MAT***a** *his3-Δ200 leu2-Δ1 lys2-801 trp1-Δ63 ura3-52*	(Bi and Pringle, 1996)
HPY16[*]	*MAT***a** *ura3-52 trp1-Δ63 leu2 his3-Δ1 pep4-3*	(Park et al., 1993)
BY4741[@]	*MAT***a** *his3Δ1 leu2Δ0 met15Δ0 ura3Δ0*	Open Biosystem
HPY1445[@]	*MAT***a** *AXL2-TAP-HIS3*	Open Biosystem
HPY1454[@]	*MAT***a** *bud3Δ::URA3 AXL2-TAP-HIS3*	This study
HPY2446[*]	*MAT***a** *bud3Δ::HIS3*	This study
HPY3130[*]	*MAT***a** *AXL2-TAP-HIS3*	This study
HPY3119[*]	*MAT***a** *BUD3-Myc*$_{13}$*-KAN AXL2-TAP-HIS3*	This study
HPY3131[*]	*MAT***a** *bud3Δ::URA3 bud3ΔN(aa 2-260)-Myc*$_{13}$*-KAN-LEU2 AXL2-TAP-HIS3*	This study
HPY3195[*]	*MAT***a** *bud4Δ::LEU2 BUD3-Myc*$_{13}$*-KAN AXL2-TAP-HIS3*	This study
HPY3196[*]	*MAT***a** *axl1::URA3 BUD3-Myc*$_{13}$*-KAN AXL2-TAP-HIS3*	This study
HPY3638[@]	*MATα CDC42-LEU2 AXL2-GFP-KAN*	This study
HPY3639[@]	*MATα cdc42-101(K5A)-LEU2 AXL2-GFP-KAN*	This study
HPY2580[@]	*MAT***a** *CDC42-LEU2 GIC2-PBD(W23A)-tdTomato-URA3 CDC3-GFP-LEU2*	This study
HPY2578[@]	*MAT***a** *cdc42-101(K5A)-LEU2 GIC2-PBD(W23A)- tdTomato-URA3 CDC3-GFP-LEU2*	This study
HPY3377[#]	*MAT***a** *CDC10-GFP-HIS3*	This study
HPY3419[#]	*MAT***a** *axl2Δ::KAN CDC10-GFP-HIS3*	This study
HPY3652[#]	*MAT***a** *AXL2-V*$_{N}$*-HIS3 CDC10-V*$_{C}$*-TRP1*	This study
HPY3641[#]	*MAT***a**/*MATα CDC10-V*$_{N}$*-HIS3/CDC10-V*$_{C}$*-TRP1*	This study
HPY3903[#]	*MAT***a**/*MATα CDC10-V*$_{N}$*-HIS3/CDC10-V*$_{C}$*-TRP1 axl2Δ::KAN/ axl2Δ::KAN*	This study
HPY3844[#]	*MATα CDC3-V*$_{N}$*-TRP1 CDC10-V*$_{C}$*-HIS3*	This study [b]
HPY3908[#]	*MAT***a** *CDC3-V*$_{N}$*-TRP1 CDC10-V*$_{C}$*-HIS3 axl2Δ::KAN*	This study [b]
HPY3915[#]	*MAT***a** *AXL2-V*$_{N}$*-HIS3 CDC11-V*$_{C}$*-TRP1*	This study [b]

HPY3912#	*MAT***a** *AXL2-V_N-HIS3 cdc11*(Δ357–415)-*V_C-TRP1*	This study [b]
HPY3917#	*MAT***a** *CDC11-tdTomato-NAT*	This study
HPY3930#	*MAT***a** *CDC11-tdTomato-NAT axl2Δ::HIS3*	This study
HPY3935	*MAT***a** *CDC3-GFP-LEU2*	This study
HPY3932#	*MAT*α *CDC3-GFP-LEU2 axl2Δ::HIS3*	This study
HPY3727#	*MAT***a** *CDC3-mCherry-LEU2 CDC10-GFP-HIS3*	This study
HPY3729#	*MAT***a** *axl2Δ::KAN CDC3-mCherry-LEU2 CDC10-GFP-HIS3*	This study

[a] Strains marked with # are congenic to YEF47A (Bi and Pringle, 1996); strains marked with @ are derived from DDY1301 or DDY1304 (in S288C background); and strains marked with * are isogenic to HPY16 (Park et al., 1993), except as indicated.

[b] Derived using YO802 (*CDC12-V_C::KanMX6),* YO685 (*CDC3-V_C::HIS3MX6),* YEF5692 *(CDC12-V_N::TRP1)*, and YEF5689 *(CDC3-V_N::TRP1)* (Oh et al., 2013).

[c] A standard PCR-mediated tagging of *V_C::TRP1* at the C terminus of Cdc11 was performed using pFA6a-VC-TRP1with the primers: oCDC112 (5'-GAGAAAGAAGAAATAAGTGAGGAAGCCAAAAGCGG ACTCAGAATTCGAGCTCGTTTAAAC-3')and oCDC113 (5'-AGCCAGGTTGGAAAAAGAGGCG AAAATCAAACAGGAAGAACGGATCCCCGGGTTAATTAA-3')

[d] A standard PCR-mediated tagging of *V_C::TRP1* at the C terminus of Cdc11 was performed using pFA6a-VC-TRP1with the primers: oCDC112 (5'-GAGAAAGAAGAAATAAGTGAGGAAGCCAAAAGCGG ACTCAGAATTCGAGCTCGTTTAAAC-3')and oCDC111(5'-AGCATCCGATATGCACGGGCAA AGCACTGGCGAAAATAACCGGATCCCCGGGTTAATTAA-3')

[e] A standard PCR-mediated tagging at the C terminus of Cdc11 was performed using pFA6a-tdTomato-NAT as a template with the primers oCDC112 and oCDC113.

Table S2. Plasmids used in this study

Plasmid name	Description	Source
pEG202	P_{ADH1}-LexA, 2μ, *HIS3*	(Gyuris et al., 1993)
pEG-cdc42^{C188S}	*cdc42^{C188S}*, pEG202	(Butty et al., 2002)
pEG-cdc42$^{G12V, C188S}$	*cdc42$^{G12V, C188S}$*, pEG202	(Butty et al., 2002)
pEG-cdc42$^{D118A, C188S}$	*cdc42$^{D118A, C188S}$*, pEG202	(Butty et al., 2002)
pEG-cdc42$^{G15A, C188S}$	*cdc42$^{G15A, C188S}$*, pEG202	This study
pEG-cdc42$^{K5A, C188S}$	*cdc42-101$^{K5A, C188S}$*, pEG202	This study
pJG4-5	P_{GAL1}-B42AD, 2μ, *TRP1*	(Gyuris et al., 1993)
pJG-Axl2(C)	*AXL2* C-terminal domain (aa 529 - 823), pJG4-5	This study
pJG-Bud3(m)	*bud3(aa 1- 656),* pJG4-5	This study
pEG-CDC3	*CDC3*, pEG202	(Farkasovsky et al., 2005)
pEG-CDC10	*CDC10*, pEG202	(Farkasovsky et al., 2005)
pEG-CDC11	*CDC11*, pEG202	(Farkasovsky et al., 2005)
pEG-CDC12	*CDC12*, pEG202	(Farkasovsky et al., 2005)
YIplac128-CDC3-GFP	*CDC3-GFP*, integrative, *LEU2*	(Gao et al., 2007)
pEGKT-CDC42	P_{GAL1}-GST-*CDC42*, 2μ, *URA3*	(Gao et al., 2007)
pRD56	P_{GAL1}-GST, 2μ, *URA3*	(Park et al., 1993)
pFA6a-VC-TRP1	For C-terminal tagging of VC, *TRP1*	(Sung and Huh, 2007)
pFA6a-tdTomato-natMX	For C-terminal tagging of tdTomato, *natMX6*	A gift from F. Chang

References

Bi, E. and Pringle, J. R. (1996). *ZDS1* and *ZDS2*, genes whose products may regulate Cdc42p in *Saccharomyces cerevisiae*. *Mol. Cell. Biol.* **16**, 5264-5275.

Butty, A.-C., Perrinjaquet, N., Petit, A., Jaquenoud, M., Segall, J. E., Hofmann, K., Zwahlen, C. and Peter, M. (2002). A positive feedback loop stabilizes the guanine-nucleotide exchange factor Cdc24 at sites of polarization. *EMBO J.* **21**, 1565-1576.

Farkasovsky, M., Herter, P., Voss, B. and Wittinghofer, A. (2005). Nucleotide binding and filament assembly of recombinant yeast septin complexes. *Biol. Chem.* **386**, 643-56.

Journal of Cell Science • Supplementary information

Gao, X. D., Sperber, L. M., Kane, S. A., Tong, Z., Tong, A. H., Boone, C. and Bi, E. (2007). Sequential and distinct roles of the cadherin domain-containing protein Axl2p in cell polarization in yeast cell cycle. *Mol. Biol. Cell* **18**, 2542-60.
Gyuris, J., Golemis, E., Chertkov, H. and Brent, R. (1993). Cdi1, a human G1 and S phase protein phosphatase that associates with Cdk2. *Cell* **75**, 791-803.
Kozminski, K. G., Chen, A. J., Rodal, A. A. and Drubin, D. G. (2000). Functions and functional domains of the GTPase Cdc42p. *Mol. Biol. Cell* **11**, 339-54.
Oh, Y., Schreiter, J., Nishihama, R., Wloka, C. and Bi, E. (2013). Targeting and functional mechanisms of the cytokinesis-related F-BAR protein Hof1 during the cell cycle. *Mol. Biol. Cell* **24**, 1305-20.
Park, H.-O., Chant, J. and Herskowitz, I. (1993). *BUD2* encodes a GTPase-activating protein for Bud1/Rsr1 necessary for proper bud-site selection in yeast. *Nature* **365**, 269-74.
Sung, M. K. and Huh, W. K. (2007). Bimolecular fluorescence complementation analysis system for in vivo detection of protein-protein interaction in Saccharomyces cerevisiae. *Yeast* **24**, 767-75.

Journal of Cell Science • Supplementary information
