# Supplementary material for: Cdc42 couples septin recruitment to the axial landmark assembly via Axl2 in budding yeast
Source: J Cell Sci. 2023 Oct 9;137(5):jcs261080. doi: 10.1242/jcs.261080 (PMC10617600; doi:10.1242/jcs.261080)
Supplement: Supplementary file 1 [file joces-137-261080_review_history.pdf]

Journal of Cell Science

Cdc42 couples septin recruitment to the axial landmark assembly via Axl2 in budding yeast

Pil Jung Kang, Rachel Mullner, Kendra Lian and Hay-Oak Park
DOI: 10.1242/jcs.261080

Editor: David Bryant

Review timeline
Original submission: 19 February 2023
Editorial decision: 27 March 2023
First revision received: 17 August 2023
Accepted: 11 September 2023

Original submission

First decision letter

MS ID#: JOCES/2023/261080

MS TITLE: Cdc42 couples septin recruitment to the axial landmark assembly via Axl2 in budding yeast

AUTHORS: Pil Jung Kang, Rachel Mullner, Kendra Lian, and Hay-Oak Park
ARTICLE TYPE: Short Report

We have now reached a decision on the above manuscript. To see the reviewers' reports and a copy of this decision letter, please go to: https://submit-jcs.biologists.org and click on the 'Manuscripts with Decisions' queue in the Author Area. (Corresponding author only has access to reviews.)

As you will see, all three reviewers gave very favourable reports but raised some critical points that will require amendments to your manuscript, and possibly some additional experimental work. I hope that you will be able to carry these out because I would like to be able to accept your paper, depending on further comments from reviewers.

Please ensure that you clearly highlight all changes made in the revised manuscript. Please avoid using 'Tracked changes' in Word files as these are lost in PDF conversion.

I should be grateful if you would also provide a point-by-point response detailing how you have dealt with the points raised by the reviewers in the 'Response to Reviewers' box. Please attend to all of the reviewers' comments. If you do not agree with any of their criticisms or suggestions please explain clearly why this is so.

Reviewer 1

Advance summary and potential significance to field

Kang, et al describe compelling genetic interactions and localization phenotypes that support a role for Axl2 in direct recruitment of septins to the bud site. It’s a short (very well-written) paper with not a lot of data but I think the data are mostly clear and support the conclusions made, with a few exceptions described below.

© 2023. Published by The Company of Biologists under the terms of the Creative Commons Attribution License (https://creativecommons.org/licenses/by/4.0/).

Comments for the author

In the two-hybrid assays, is DBD fused to each septin's N terminus, or C terminus?
Cdc10 is unique among the septins in lacking an extended, flexible C terminal tail. If the DBD fusions are C-terminal, and Axl2 interacts with the globular domain of all septins, then it seems possible that Cdc10 might appear to be the only Axl2-interacting septin simply because only for Cdc10 is the DBD domain in close enough proximity to the AD domain fused to Axl2. If the DBD is fused to each septin's N terminus, then only for Cdc3 would there be an issue of interpretation since only Cdc3 has a long, flexible N-terminal extension. The BiFC fusions are definitely C-terminal, and Cdc12 has a long, flexible C-terminal tail. Thus again the reason Cdc10 might seem to be the direct binder of Axl2 could be this tag proximity issue. This could be addressed by inserting something like CFP in between Cdc10 and the DBD or BiFC tag, to mimic the "separation" that is seen in other septins, or, alternatively, delete the C-terminal tail of Cdc11 (for example) in a fusion and see if it now starts to show Axl2 interaction. N-terminal fusions would also partly alleviate concerns.

The interpretation of the BiFC signals in Figure 4 is problematic. It seems more likely to me that the lack of axl2Δ simply perturbs septin recruitment to a given cortical site, which decreases local signal, but does not perturb the Cdc3–Cdc10 interaction. On their own, the BiFC tags cause slight but significant functional septin defects, and combined with the axl2Δ this may simply reduce the efficiency of preassembled septin complex recruitment. The Cdc10-GFP defects noted in axl2Δ cells in Fig. S3 compared to apparently normal localization of Cdc3-GFP in those cells could simply reflect more severe functional impact of the GFP tag on Cdc10 compared to Cdc3.

The authors acknowledge some of these caveats in their "Limitations" section which is good, but it comes a little late after the relatively strong wording that accompanies the description of the results.

If Axl2 binds Cdc10 and only Cdc10 (among septins), then is it completely dispensable for septin localization in a cdc10Δ background? Alternatively, if Axl2 overexpression suppresses the lethality of spa2Δ cdc10-10, to me this suggests that it acts independently of Cdc10, since the cdc10-10 allele carries a premature stop at codon 133 and is likely a null allele. It would be important to test the phenotype of combining axl2Δ with cdc10Δ and of Axl2 overexpression in a cdc10Δ background.

Reviewer 2

Advance summary and potential significance to field

See attached pdf.

Comments for the author

The manuscript "Cdc42 couples septin recruitment to the axial landmark assembly via Axl2 in budding yeast" is a short report that examines how a cell links a spatial cue to septins, proteins necessary for proper cytokinesis. Using data from live-cell imaging, two-hybrid assays, and mutant analysis, the authors propose that the Rho GTPase Cdc42, when GTP bound, interacts with the landmark protein Axl2, to facilitate septin recruitment to the future site of cytokinesis.

Although this is a short study, it is solidly focused on deciphering mechanism. Another plus of this study is that it reveals at least one defect of a workhorse temperature-sensitive *cdc42* allele. As part of that mutant analysis, the authors have presented the spatio-temporal distributions of several proteins of functional significance that interact with Cdc42. Glimpses of these localizations have been spotted in both published and unpublished work for almost the past 25 years, often in fixed cells. It is good to see a coherent report of these localizations in live cells. These data will be useful to many yeast labs that study cell polarization, polarized cell growth, and cytokinesis.

A weakness of this study is hedging. The spatio-temporal relationships among the proteins under study are not always clear, in the sense that localization may precede downstream activation or an

© 2023. Published by The Company of Biologists under the terms of the Creative Commons Attribution License (https://creativecommons.org/licenses/by/4.0/).

activation event requires multiple inputs. In these cases, the authors state that proteins of interest "guide" other proteins. Does "guide" mean recruit, partially recruit, is a pre-requisite for assembly, or is a lead co-traveler? I understand the dilemma the authors face because they are looking at an orchestra of molecular relationships. This weakness should not be considered a dealbreaker for publication because the data within the manuscript is of value. However, the authors need to consider phrasing their conclusions using existing terminology, so far as possible.

The authors also need to be more explicit in contrasting new and old models. For example, in the introduction, the authors state Axl2 guides Cdc42, but then on page 4 (line 18) conclude that Cdc42 guides Axl2. This is a big turnaround in thinking. Briefly contextualizing new conclusions with respect to what is "known" will improve the manuscript for readers who do not regularly think about the temporal dynamics of these proteins and protein modules.

Additional Comments

1. Page 2, line 41. "Cdc42" should read "*CDC42.*"
2. Page 3, line 23. Add "cells" after *bud3 delta*."
3. Page 3, line 31. This sentence is awkward.
4. Figure 1A, third panel. There is a substantial amount of background signal on the blot which is distracting. Recommend cropping this panel to eliminate the background.
5. Figure 2B. i) Red arrows on black backgrounds are invisible to many who are color blind. Recommend changing the color of the arrow. ii) It is unclear what is measured on the y-axis of the box graph. Relative ratio of [Cdc42-GTP]. Relative to what?
6. Figure 3B. The strain containing Cdc12-Vc is not a good control for this experiment because it does not have a wild-type morphology. Is the absence of an observable interaction due to the fact that Axl2 and Cdc12 do not normally interact or due to the fact that they cannot interact in these morphologically perturbed cells? Might Cdc3-Vc or Cdc11-Vc serve as a better control?
7. Page 5, line 25. "impact" should be "impacts"
8. Page 7, line 6. What % agarose is the agarose slab?
9. Figure S1. The Axl2-Myc blot for the GST-pull down is not publication quality.
10. References. Consider including Caviston *et al. Mol Biol Cell* (2003).

Reviewer 3

Advance summary and potential significance to field

The manuscript "Cdc42 couples septin recruitment to the axial landmark assembly via Axl2 in budding yeast" by Kang et al. investigates how septins are recruited to the axial bud site. The authors show that Axl2 interacts with Bud3 and GTP-bound Cdc42, in addition to the septin subunit Cdc10. They used a temperature-sensitive cdc42 mutant which is defective for interaction with Axl2 and showed that in this mutant septin recruitment in G1 is compromised. Together these results indicate that active Cdc42 is critical for localizing Axl2 (recruiting or stabilizing its localization) which is turn plays a key role in septin recruitment to the axial bud site. Overall, this work is convincing and clearly described. The data are of high quality and this work will be of interest to a general audience.

Comments for the author

Below I have indicated some minor issues that should be easily addressable:

1) The cdc42-101 mutant phenotype and its defect in interaction with Axl2 carboxy-terminus by two-hybrid are convincing, however it would be important to show that the interaction of this Rho GTPase mutant with other effectors/partners are unaffected. If this was done in the Kozminski 2000 or Kang 2014 papers, this should be stated.

2) Abstract (page2) lines 12-14, this sentence is unclear:

© 2023. Published by The Company of Biologists under the terms of the Creative Commons Attribution License (https://creativecommons.org/licenses/by/4.0/).

“Interestingly, a temperature-sensitive cdc42 mutant defective in the axial budding pattern at a semi-permissive temperature poorly interacted with Axl2 and exhibited compromised septin recruitment in the G1 phase.”
and should be changed to:
Interestingly, a cdc42 mutant defective in the axial budding pattern at the semi-permissive temperature had a reduced interaction with Axl2 and compromised septin recruitment in the G1 phase.

3) In two places in the text, percentages are indicated, yet n should also be indicated (it is not); see lines 27 and 28, page 4.

4) Figure 3A, is the interaction of Axl2 with Cdc10 depend upon GTP binding of septin, i.e. does it occur with a GTP binding-defective septins?

5) Page 5, line 29. It is not clear what “loosely organized” means, please be more precise and/or quantitative with respect to what this means or what is observed.

6) In the pull down panles, Figures 1A and S1 quantitation would improve these observations, i.e. to show the fold/% decrease of Bud3 associated with Axl2 in the two mutants (axl1 and bud3Δ), as well as with the bud3 truncation. Similarly in Figure S1 Axl2 GST-pull down appears to increase in the bud3Δ strain. It should also be indicated in the figure legend how many pull down experiments were carried out and standard deviation for quantitation, if relevant.

7) Figure 2B, for comparison, it would be helpful to include the wild-type strain (relevant to the quantitation graph). In the graph, the n can be moved to the figure legend. Also the y-axis indicates “relative ratio” however it needs to be stated what is the ratio of?

8) Figure 3B, the split YFP scheme can be removed, I think most people understand how BiFC works.

First revision

Author response to reviewers' comments

Please note: we have uploaded this as Supplementary Information.

Reviewers’ comments are shown in italics, and our responses are shown after each comment. All modified text is highlighted in yellow.

Reviewer 1 Advance summary and potential significance to field...
Kang, et al describe compelling genetic interactions and localization phenotypes that support a role for Axl2 in direct recruitment of septins to the bud site. It’s a short (very well-written) paper with not a lot of data but I think the data are mostly clear and support the conclusions made, with a few exceptions, described below.

Reviewer 1 Comments for the author...
(Numbers are inserted for each comment’s organization.)

1. In the two-hybrid assays, is DBD fused to each septin’s N terminus, or C terminus? Cdc10 is unique among the septins in lacking an extended, flexible C terminal tail. If the DBD fusions are C-terminal, and Axl2 interacts with the globular domain of all septins, then it seems possible that Cdc10 might appear to be the only Axl2-interacting septin simply because only for Cdc10 is the DBD domain in close enough proximity to the AD domain fused to Axl2. If the DBD is fused to each septin’s N terminus, then only for Cdc3 would there be an issue of interpretation, since only Cdc3 has a long, flexible N-terminal extension.

© 2023. Published by The Company of Biologists under the terms of the Creative Commons Attribution License (https://creativecommons.org/licenses/by/4.0/).

– DBD is fused to the N terminus of each septin subunit. The distances between the DBD domain of each septin fusion and the AD domain fused to Axl2 would not be significantly different, except for Cdc3. The N-terminal region of Cdc3 has about 86 ~ 98 residues longer (from the common phosphoinositide interaction domain and the globular GTPase domain) than other subunits. We think this difference is unlikely to impact the outcomes of the two-hybrid assay. Although DBD and AD should be brought in proximity to turn on the transcription of a reporter gene, the distance between DBD (which binds the promoter of a reporter gene) and AD (which interacts with the transcription machinery, including RNA pol II) would not be as stringent as in a BiFC assay, which requires tight proximity between YFP-N and YFP-C for the structural complementation of YFP reconstitution.

2. The BiFC fusions are definitely C-terminal, and Cdc12 has a long, flexible C-terminal tail. Thus again the reason Cdc10 might seem to be the direct binder of Axl2 could be this tag proximity issue. This could be addressed by inserting something like CFP in between Cdc10 and the DBD or BiFC tag, to mimic the “separation” that is seen in other septins, or, alternatively, delete the C-terminal tail of Cdc11 (for example) in a fusion and see if it now starts to show Axl2 interaction. N-terminal fusions would also partly alleviate concerns.

– We thank the reviewer for this suggestion and agree with the reviewer that the proximity of VN and VC tags affects the positive interaction in BiFC assays. Since VC is fused to the C terminus of each septin, the difference in the C-terminal extension (CTE) might have potentially skewed the outcomes if a central domain common to all septin subunits was involved in the interaction with Axl2. Since a previous study suggested that Cdc11 CTE is dispensable for its function, unlike Cdc3 and Cdc12 (Versele et al., MBoC 2004 [PMID: 15282341]), we tested the VC fusions of Cdc11 and Cdc11ΔC, which lacks its CTE (residues 357-415).

Cdc11-VC was fully functional based on the growth phenotype and cell morphology. The expression of Cdc11ΔC-VC did not affect cell viability but caused a slightly elongated cell shape in a small percentage of cells, consistent with a previous report (Versele et al., MBoC 2004). We found no detectable YFP signal in BiFC assays when Cdc11-VC or Cdc11ΔC-VC was co-expressed with Axl2-VN, as in the case of the co-expression of Cdc12-VC and Axl2-VN. Since Cdc12-VC does not appear fully functional (based on cell morphology), we now present the BiFC data with Cdc11-VC and Cdc11ΔC-VC (see Figure 3B; lines 2–11, page 5). These results, together with two-hybrid assays, thus suggest that Axl2 interacts closely with Cdc10.

3. The interpretation of the BiFC signals in Figure 4 is problematic. It seems more likely to me that the lack of axl2Δ simply perturbs septin recruitment to a given cortical site, which decreases local signal, but does not perturb the Cdc3–Cdc10 interaction. On their own, the BiFC tags cause slight but significant functional septin defects, and combined with the axl2Δ this may simply reduce the efficiency of preassembled septin complex recruitment. The Cdc10-GFP defects noted in axl2Δ cells in Fig. S3 compared to apparently normal localization of Cdc3-GFP in those cells could simply reflect more severe functional impact of the GFP tag on Cdc10 compared to Cdc3. The authors acknowledge some of these caveats in their “Limitations” section, which is good, but it comes a little late after the relatively strong wording that accompanies the description of the results.

– The first issue is regarding the interpretation of the BiFC results. We agree with the reviewer that we cannot conclude that axl2Δ perturb the Cdc3-Cdc10 interaction based on these BiFC data. We thus modified the subheading and text (line 13 & lines 38–39, page 5).

– The second issue is regarding the functionality of Cdc10 tags. We did not observe any obvious growth defect or abnormal cell morphology of the strains expressing Cdc10-tagged with GFP (or VN or VC) at its C-terminus, consistent with previous reports (e.g., Weems et al., eLife 2017 [PMID: 28541184]; McMurray et al., Dev Cell 2011 [PMID: 21497764]). Surprisingly, however, we found that Cdc3-mCherry localizes to the bud tip in some axl2Δ CDC10-GFP cells, even though such abnormal localization of Cdc3-GFP was not observed in the axl2Δ CDC10 (untagged) cells. These observations suggest that GFP-tagging of Cdc10 causes a subtle defect, which is exacerbated in combination with axl2Δ. We presented these in Figure S3, C and discussed in text (lines 27–35, page 5).

4. If Axl2 binds Cdc10 and only Cdc10 (among septins), then is it completely dispensable for septin localization in a cdc10Δ background? Alternatively, if Axl2 overexpression suppresses the lethality

© 2023. Published by The Company of Biologists under the terms of the Creative Commons Attribution License (https://creativecommons.org/licenses/by/4.0/).

of spa2Δ cdc10-10, to me this suggests that it acts independently of Cdc10, since the cdc10-10 allele carries a premature stop at codon 133 and is likely a null allele. It would be important to test the phenotype of combining axl2Δ with cdc10Δ and of Axl2 overexpression in a cdc10Δ background.

- We agree with the reviewer that cdc10-10 is likely a null allele, but we cannot easily reconcile our findings (listed below) with the previous genetic data that overexpression of AXL2 suppresses spa2Δ cdc10-10 (Roemer et al., 1996). We noted similar genetic puzzles or ambiguities previously. For example, bud2Δ is synthetic-lethal with cln1Δ cln2Δ (Cvrcková & Nasmyth, EMBO J 1993 [PMID: 8262070]), suggesting that Bud2 functions in a pathway parallel to the G1 cyclin Cln1 or Cln2. Yet BUD2 exhibits a two-hybrid interaction with CLN2 (Drees et al., J Cell Biol 2001 [PMID: 11489916]), and Bud2 is likely a substrate of Cdc28-Cln1/2 (Holt et al., Science 2009, [PMID: 19779198]; Faustova et al., Int J Mol Sci 2021, [PMID: 34502421]), consistent with the temporal regulation of bud-site selection and polarity establishment.

As suggested, we examined the localization of Cdc3 and Cdc11 in cdc10Δ and axl2Δ cdc10Δ cells and found that both Cdc3 and Cdc11 localize similarly at least in those cdc10Δ and axl2Δ cdc10Δ cells that had cell shapes similar to WT cells at ~ 24°C (although these cells were somewhat larger than WT cells and had ovoid shapes). However, we also observed very heterogeneous, abnormal cell shapes and frequent occurrence of a suppressor mutation (or gene conversion of cdc10Δ) in these cdc10Δ strains after passaging them at room temperature. It is currently unclear whether the sickness of these strains (even with a cdc10Δ single mutation) is due to any additive effect of tagging of Cdc3 or Cdc11 in these cdc10Δ strains (despite no obvious defects or abnormal cell shapes in the CDC3-GFP or CDC11-tdTomato strains in otherwise WT background). It is also unclear whether axl2Δ exacerbates the sickness of these tagged cdc10Δ strains. To avoid potential misrepresentation using a subset of cells with more normal cell shapes, we decided not to present septin localization in these cdc10Δ and axl2Δ cdc10Δ cells until further investigation.

Despite these limitations, our current data suggest that Axl2 is involved in septin recruitment via its interaction with Cdc10 as follows:
1) In a two-hybrid assay, Axl2 interacts with Cdc10 but not with Cdc3, Cdc11, or Cdc12 (Fig. 3A).
2) In BiFC assays, Axl2 interacts with Cdc10 but not with Cdc11, Cdc11ΔCTE, or Cdc12 (Fig. 3B & data not shown; see above, #2 & #3).
3) Deletion of AXL2 disturbs the localization of Cdc10 (Fig. 4A) but not Cdc3 and Cdc11 (Fig. S3).

Reviewer 2 Comments for the author...
The manuscript “Cdc42 couples septin recruitment to the axial landmark assembly via Axl2 in budding yeast” is a short report that examines how a cell links a spatial cue to septins, proteins necessary for proper cytokinesis. Using data from live-cell imaging, two-hybrid assays, and mutant analysis, the authors propose that the Rho GTPase Cdc42, when GTP bound, interacts with the landmark protein Axl2, to facilitate septin recruitment to the future site of cytokinesis.

Although this is a short study, it is solidly focused on deciphering mechanism. Another plus of this study is that it reveals at least one defect of a workhorse temperature-sensitive cdc42 allele. As part of that mutant analysis, the authors have presented the spatio-temporal distributions of several proteins of functional significance that interact with Cdc42. Glimpses of these localizations have been spotted in both published and unpublished work for almost the past 25 years, often in fixed cells. It is good to see a coherent report of these localizations in live cells. These data will be useful to many yeast labs that study cell polarization, polarized cell growth, and cytokinesis.

A weakness of this study is hedging. The spatio-temporal relationships among the proteins under study are not always clear, in the sense that localization may precede downstream activation or an activation event requires multiple inputs. In these cases, the authors state that proteins of interest “guide” other proteins. Does “guide” mean recruit, partially recruit, is a pre-requisite for assembly, or is a lead co-traveler? I understand the dilemma the authors face because they are looking at an orchestra of molecular relationships. This weakness should not be considered a dealbreaker for publication because the data within the manuscript is of value. However, the authors need to consider phrasing their conclusions using existing terminology, so far as possible.

© 2023. Published by The Company of Biologists under the terms of the Creative Commons Attribution License (https://creativecommons.org/licenses/by/4.0/).

The authors also need to be more explicit in contrasting new and old models. For example, in the introduction, the authors state Axl2 guides Cdc42, but then on page 4 (line 18) conclude that Cdc42 guides Axl2. This is a big turnaround in thinking. Briefly contextualizing new conclusions with respect to what is “known” will improve the manuscript for readers who do not regularly think about the temporal dynamics of these proteins and protein modules.

– We are grateful to the reviewer for pointing out those ambiguous and confusing descriptions. Although our data are still limited for making a conclusive statement regarding the spatiotemporal relationships, we carefully revised the text, as summarized below:
1) Abstract: The sentence “Our findings suggest that active Cdc42 guides Axl2, which plays a key role in linking septin recruitment to the spatial cue at the axial bud site” is changed to “We propose that active Cdc42 brings Axl2 to the Bud3-Bud4 complex and that Axl2 then interacts with Cdc10, linking septin recruitment to the axial landmark” (lines 13-14, page 2). We avoid using ‘recruit’ here because Cdc42-GTP is unlikely to stay with the Bud3-Bud4 complex, while Bud3 and Bud4 ‘recruit’ Axl2.

2) Introduction: The previous sentence “This budding pattern depends on the axial landmark, including Axl2, which guides the Rsr1 and Cdc42 GTPase modules (Bi and Park, 2012)” is deleted. Instead, to distinguish the old model (a linear hierarchy) vs. a new model (non-linear crosstalk), these sentences are added: “Earlier studies suggested a linear morphogenetic hierarchy from spatial landmarks to the cytoskeletons via the Rsr1 and Cdc42 GTPase modules (citations). However, temporal interactions among the landmark components, Cdc42, and septins suggest more complex crosstalk between the proteins involved in polarity establishment.” (lines 23–27, page 2). We then discuss these specific interactions in the subsequent sentences.

Additional Comments
1. Page 2, line 41. “Cdc42” should read “CDC42.”
   – This is corrected.

2. Page 3, line 23. Add “cells” after bud3 delta.”
   – This is added, as suggested.

3. Page 3, line 31. This sentence is awkward.
   – The text is modified (lines 27–28, page 3).

4. Figure 1A, third panel. There is a substantial amount of background signal on the blot which is distracting. Recommend cropping this panel to eliminate the background.
– We modified the figure, as suggested. We also added the relative ratio of Bud3 (or Bud3ΔN) associated with Axl2-TAP.

5. Figure 2B. i) Red arrows on black backgrounds are invisible to many who are color blind. Recommend changing the color of the arrow. ii) It is unclear what is measured on the y-axis of the box graph. Relative ratio of [Cdc42-GTP]. Relative to what?

i) We regret this oversight. The arrow color is now changed.
ii) The polarized Cdc42-GTP cluster in time-lapse images of WT and cdc42-101 cells was quantified by a threshold method using an ImageJ macro. From these analyses at every time point, only the peak value in each time window was then plotted after normalizing against the average peak intensity in WT cells during the second time window (i.e., early G1) in Figure 2C. This threshold macro has been used in several previous studies by us and others (Okada et al., 2013, PMID: 23906065; Kang et al., 2014 PMID: 25002677; Lee et al., 2015, PMID: 25908844; Okada et al., 2017, PMID: 28336063). Details of this analysis are described in Materials and Methods with proper citations (lines 3–11, page 7).

6. Figure 3B. The strain containing Cdc12-Vc is not a good control for this experiment because it does not have a wild-type morphology. Is the absence of an observable interaction due to the fact that Axl2 and Cdc12 do not normally interact or due to the fact that they cannot interact in these morphologically perturbed cells? Might Cdc3-Vc or Cdc11-Vc serve as a better control?

© 2023. Published by The Company of Biologists under the terms of the Creative Commons Attribution License (https://creativecommons.org/licenses/by/4.0/).

– We agree with the reviewer's concern about the abnormal shapes of some Cdc12-Vc cells, although no positive BiFC signal was detected even among the Cdc12-Vc cells with normal morphology (which were more abundant). We modified Figure 3 with Cdc11-VC and Cdc11ΔC-VC (see above, Comment #2, Reviewer 1).

7. Page 5, line 25. "impact" should be "impacts"
– We thank the reviewer for careful editing; this typo is corrected.

8. Page 7, line 6. What % agarose is the agarose slab?
- '2% agarose' is added (line 39, page 6).

9. Figure S1. The Axl2-Myc blot for the GST-pull down is not publication quality.
- We replaced Figure S1 with the AXL2-TAP pull-down result. Although the percentage of Cdc42 pulled down with Axl2 is still low (< 0.2% of input, likely because of scarce Cdc42-GTP in vivo), the result unambiguously shows that Axl2 interacts with Cdc42 in WT and *bud3Δ* cells (Figure S1).

10. References. Consider including Caviston et al. Mol Biol Cell (2003).
- We regret this oversight. The reference is added, as suggested (line 22, page 2).

Reviewer 3 Advance summary and potential significance to field...
The manuscript "Cdc42 couples septin recruitment to the axial landmark assembly via Axl2 in budding yeast" by Kang et al. investigates how septins are recruited to the axial bud site. The authors show that Axl2 interacts with Bud3 and GTP-bound Cdc42, in addition to the septin subunit Cdc10. They used a temperature-sensitive cdc42 mutant which is defective for interaction with Axl2 and showed that in this mutant septin recruitment in G1 is compromised. Together these results indicate that active Cdc42 is critical for localizing Axl2 (recruiting or stabilizing its localization) which is turn plays a key role in septin recruitment to the axial bud site. Overall, this work is convincing and clearly described. The data are of high quality and this work will be of interest to a general audience.

Reviewer 3 Comments for the author...
Below I have indicated some minor issues that should be easily addressable:
1) The cdc42-101 mutant phenotype and its defect in interaction with Axl2 carboxy-terminus by two-hybrid are convincing, however it would be important to show that the interaction of this Rho GTPase mutant with other effectors/partners are unaffected. If this was done in the Kozminski 2000 or Kang 2014 papers, this should be stated.

- We are grateful to the reviewer for this suggestion. Although this has not been directly tested biochemically, some pieces of evidence suggest that the cdc42-101 mutant is not generally defective in interaction with its effectors or other binding partners. We showed that the polarized cluster of Cdc42-GTP is present at a similar level in WT and cdc42-101 cells before cytokinesis (using PBD-RFP, which specifically interacts with Cdc42-GTP; Okada et al., Meth Enzym 2017; PMID: 28336063) (see Figure 2, B & C). It is thus unlikely that Cdc42-101 is defective in interaction with its effectors, which have the PBD/CRIB domain.

This high level of PBD-RFP cluster in cdc42-101 cells (except in early G1) also suggests that the mutant is unlikely to be defective in interaction with its GEFs – Cdc24 and Bud3. Indeed, a two-hybrid assay indicates that the mutant is not defective in interaction with Bud3 (Figure 1C), although Cdc24 is not tested for its interaction with Cdc42-101. We note the low Cdc42-GTP in early G1 is not due to its poor interaction with Bud3 but compromised positive feedback regulation of Cdc42 polarization (due to poor recruitment of Axl2, which results in a failure in the interaction with the Rsr1 GTPase module and thus inefficient Cdc42 polarization: Kang et al., 2014 PMID: 25002677; Lee et al., JCS 2015 PMID: 25908844). We added additional sentences regarding these points (lines 33–38, page 4).

2) Abstract (page2) lines 12-14, this sentence is unclear:
"Interestingly, a temperature-sensitive cdc42 mutant defective in the axial budding pattern at a semi-permissive temperature poorly interacted with Axl2 and exhibited compromised septin recruitment in the G1 phase." and should be changed to:

© 2023. Published by The Company of Biologists under the terms of the Creative Commons Attribution License (https://creativecommons.org/licenses/by/4.0/).

Interestingly, a cdc42 mutant defective in the axial budding pattern at the semi-permissive temperature had a reduced interaction with Axl2 and compromised septin recruitment in the G1 phase.

- We are grateful to the reviewer for the careful editing. The abstract is modified, as suggested.

3) In two places in the text, percentages are indicated, yet n should also be indicated (it is not); see lines 27 and 28, page 4.

- Although the number of time-lapse images (n) was presented next to each strain name in Figure 2A in the initial submission, we realized this presentation might have been less clear. To be consistent with other data presentations, we now add (n) in the revised text (lines 18–19, page 4).

4) Figure 3A, is the interaction of Axl2 with Cdc10 depend upon GTP binding of septin, i.e. does it occur with a GTP binding-defective septins?

- This is indeed an important question. However, we are afraid we may not be able to complete the test within the time limit for this MS revision. In addition, because of the length limit of this short report, we hope to address this question in another study.

5) Page 5, line 29. It is not clear what "loosely organized" means, please be more precise and/or quantitative with respect to what this means or what is observed.

- We regret it was not clear with the limited number of cell images presented. We modified the text to "The YFP signals at the cell division site often appeared as a less tightly organized ring in axl2Δ cells than in WT cells (see Materials and Methods)" (lines 28–29, page 5). Because of the length limit of the report, we provided additional details in Materials and Methods (lines 29-32, page 7).

6) In the pull down panles, Figures 1A and S1 quantitation would improve these observations, i.e. to show the fold/% decrease of Bud3 associated with Axl2 in the two mutants (axl1 and bud3Δ), as well as with the bud3 truncation. Similarly in Figure S1 Axl2 GST-pull down appears to increase in the bud3Δ strain. It should also be indicated in the figure legend how many pull down experiments were carried out and standard deviation for quantitation, if relevant.

- We regret this oversight. We included the relative amount of Bud3 associated with Axl2 (see Figure 1A). To examine the interaction between Axl2 and Cdc42 in WT vs bud3Δ, we repeated two different ways – either pulling-down GST-Cdc42 or Axl2-TAP. Although we see a slightly higher association of these proteins in bud3Δ cells on average (Figure S1), the physiological significance of this minor difference is not clear. Additional details are provided in Materials and Methods (lines 18–23, page 8).

7) Figure 2B, for comparison, it would be helpful to include the wild-type strain (relevant to the quantitation graph). In the graph, the n can be moved to the figure legend. Also the y-axis indicates "relative ratio" however it needs to be stated what is the ratio of?
- We are grateful to the reviewer for this suggestion, and the WT images are now added in Figure 2B. Although similar time-lapse images of WT have been shown in our previous publications, this strain background is different from the WT images used in those previous studies.
- The fluorescence intensity of PBD-RFP was quantified at each time lapse image using a threshold method in ImageJ macro. The peak intensities at each time window were plotted after being normalized by the average peak value of WT in early G1 (time window 2) (see details in Materials & Methods; lines 3-11, page 7).

8) Figure 3B, the split YFP scheme can be removed, I think most people understand how BiFC works.

- The scheme is removed, as suggested.

© 2023. Published by The Company of Biologists under the terms of the Creative Commons Attribution License (https://creativecommons.org/licenses/by/4.0/).

Second decision letter

MS ID#: JOCES/2023/261080

MS TITLE: Cdc42 couples septin recruitment to the axial landmark assembly via Axl2 in budding yeast

AUTHORS: Pil Jung Kang, Rachel Mullner, Kendra Lian, and Hay-Oak Park
ARTICLE TYPE: Short Report

I am delighted to tell you that your manuscript has been accepted for publication in Journal of Cell Science, pending standard ethics checks. I do note that Reviewer 3 has picked up a potential typographical error. If you could please answer this query, we will have it updated in the manuscript.

Reviewer 1

Advance summary and potential significance to field

This work provides new insights into coupling of cell polarity landmarks to the polarity machinery and downstream effectors. It clarifies some uncertainties in the field and adds new relationships between established molecular players that significantly advance our understanding.

Comments for the author

I am satisfied with the authors' revisions to the manuscript and responses to my comments and to the comments of the other reviewers.
I note with regard to Reviewer #3's comment #4 about whether the interaction of Axl2 with Cdc10 depends upon GTP binding of septin: I think this question would unfortunately, be difficult to address using septin mutants with substitutions in residues that contact bound nucleotide. It has become increasingly clear that almost every residue that directly contacts bound nucleotide also has the potential to contact (directly or indirectly) another septin across the G dimerization interface, making it very difficult to interpret phenotypes (UNLESS of course there is no phenotype!). Thus nucleotide binding and G dimerization are almost inextricably linked, from an experimental standpoint.

Reviewer 2

Advance summary and potential significance to field

As described in my initial review.

Comments for the author

The authors satisfactorily addressed all of the the concerns contained in my initial review.

Reviewer 3

Advance summary and potential significance to field

The authors have satisfactorily addressed all of the reviewers concerns.

Comments for the author

In Figure S1 it now itndictates % of GST-Cdc42 pulled down relative to the input. It indicates 0.16 and 0.18 for WT and bud3delta, respectively. I am guessing the authors meant either 0.16/0.18 relative to 1 or 16% and 18% but not 0.16 and 0.18%?

© 2023. Published by The Company of Biologists under the terms of the Creative Commons Attribution License (https://creativecommons.org/licenses/by/4.0/).

Second revision

Author response to reviewers' comments

Regarding the reviewer #3's comment, I confirm that the numbers in our Figure S1 is correct (i.e., 0.16 and 0.18%, not 16 and 18%). As indicated in our rebuttal letter, the percentage of Cdc42 pulled down with Axl2 is very low (< 0.2% of input), likely because of scarce Cdc42-GTP in vivo (see our response to Reviewer #2, #9). Although it appears a higher percentage on the immunoblot, please note that the pull-down samples were more concentrated compared to the input for immunoblotting. We then calculated the relative recovery."

© 2023. Published by The Company of Biologists under the terms of the Creative Commons Attribution License (https://creativecommons.org/licenses/by/4.0/).
